# Parliament and the Professions

**Published:** 1920-06-19

**Authors:** 


### Parliament and the Professions.

### National Hospital for the Paralysed and Epileptic.

SIR CLEMENT KINLOCH-COOKE asked the Minister of Health whether he was aware that the National Hospital for the Paralysed and Epileptic, the principal school for the study of nervous diseases, has had to close its doors owing to want of funds, and, in view of the urgent public need of keeping the hospital open, and the serious loss to medical science involved, could he now make the promised statement with regard to Government assistance to hospitals. Dr. Addison replied that he was not yet in a position to make any general statement, but, in the meantime, he understood that King Edward's Hospital Fund are prepared to consider the application from this hospital for an immediate emergency grant which had reached them, no previous intimation having been given to the Fund that there was any intention of closing the hospital. Sir C. Kinloch-Cooke reminded Dr. Addison that he had put a question on similar lines three weeks previously, and had then stated that the hospital would have to be closed if a grant were not forthcoming.

### Operations at Sea in Wartime.

DR. CHEVALIER, the General Medical Inspector of the Public Health and Marine Service of the United States, has issued a report on the part played by hospital ships during the war. He states, although it seems hardly necessary, that these ships were used exclusively as a means of evacuating the sick and wounded. They aided in transporting the wounded of the Belgian Army, and brought home 220,000 casuals from the various Mediterranean fronts in the East. While on the ships the men were well fed and quartered and received excellent medical care. The rocking of the boat proved only a slight disadvantage, and it was even possible to do a great amount of surgical work. The statistics show that one surgeon during thirty-nine days of hospitalisation performed 155 major operations and treated 1,884 wounded; and another, in fifteen crossings, performed 968 operations, 300 of which were under general anæsthesia.

